# Factors Associated with Overutilization of Computed Tomography Cervical Spine Imaging

**DOI:** 10.5811/westjem.18570

**Published:** 2024-02-28

**Authors:** Tessy La Torre Torres, Jonathan McGhee

**Affiliations:** ChristianaCare Hospital, Department of Emergency Medicine, Newark, Delaware

Dear Editor:

We are writing to provide some comments on the scientific paper recently published in your journal titled “Factors Associated with Overutilization of Computed Tomography of the Cervical Spine.”^1^

Firstly, we commend the authors for putting together a relevant and well-done multicenter study that both revalidates the NEXUS criteria and offers insight into the overutilization of computed tomography (CT) for traumatic injuries. However, we do have some concerns about the methodology used. Having a single reviewer collecting chart data on NEXUS criteria—criteria that we know include the subjective component of a distracting injury or deficit not attributable to pain—introduces the possibility of bias; it would, therefore, be beneficial to see congruence of chart analysis between different reviewers. It is also recognizable that there were timing constraints related to feasibility, thus allowing for only one person to review each chart for the presence of NEXUS criteria. The process involved combing through more than 800 individual records that included physician documentation, imaging, lab studies, and nursing notes. This added significantly to the workload of the single reviewer, which could have impacted the overall accuracy of the data collected. Additionally, it was unclear whether the reviewer was blind to the result of the CT when reviewing the chart, opening up further opportunities for bias.

Secondly, the short time frame in this case linked to skiing/winter sports-related injuries may provide only a partial picture, limiting the applicability of results. Imagine the study had been conducted for longer than two months outside the winter season. Would there be additional variables regarding the mechanism of injury associated with the overutilization of CT imaging not otherwise uncovered in their initial review? Additionally, the baseline characteristics for the presenting mechanism of injury included falls, which constituted approximately 75% of the total number. Further characterization of the mechanism of injury may also have been beneficial—fall from standing vs from a height, or motor vehicle collision with airbag deployment vs without— could all reveal associations of injury that would cause physicians to bypass the NEXUS criteria altogether.

Future studies should look to investigate whether a physician-in-triage structure is associated with increased CT overutilization. We are seeing more protocols being implemented in emergency medicine, including within the triage process, and it would, therefore, be interesting to see how this alternate workflow would affect results.

Overall, we found the authors’ study to be extremely informative, and we appreciate their contribution to the ever evolving and highly challenging field of emergency medicine.
